# Bridging the gap between hepatocellular carcinoma management guidelines and personalised medicine: a Bayesian network study

**DOI:** 10.3389/fbinf.2025.1574797

**Published:** 2025-05-29

**Authors:** Yi-Chun Wang, Daniel Bulte, Michael Brady

**Affiliations:** ^1^ Department of Engineering Science, Institute of Biomedical Engineering, University of Oxford, Oxford, United Kingdom; ^2^ Perspectum Ltd., Oxford, United Kingdom

**Keywords:** Bayesian network, BCLC, causal inference, counterfactual reasoning, HCC, individualised treatment effect, liver cancer, guideline

## Abstract

**Introduction:**

There are numerous treatment options available for patients with confirmed hepatocellular carcinoma (HCC). Guidelines such as Barcelona Clinic Liver Cancer (BCLC) support treatment decisions by way of a flow diagram that is organized around groups of patients. Though such guidelines continue to make a major contribution to standardization of treatment, in clinical reality, cases are often more nuanced than is captured in any flow diagram, even one as comprehensive as BCLC. A fundamental challenge for a clinician is to combine such a population-wide guideline with specific information about the individual patient. Bayesian networks (BNs) offer a way to “bridge this gap” and combine standardized care and precision medicine. They do this by enabling answers to detailed “what-if” questions from the clinician.

**Methods:**

We use real-world data of HCC patients who received treatments between 2019 and 2020 to construct a BN to assess the potential treatment effect for cases that were ***not*** treated in compliance with BCLC.

**Results:**

We report detailed scenarios for ten randomly selected cases and summarise the difference in survival time for each scenario. For each case, the counterfactual treatment scenarios are made based on whether or not the case is in compliance with BCLC guidelines, the type of treatment received and the waiting time to receive treatment.

**Discussion:**

We consider two cases with similar clinical characteristics (but received different treatments) and discuss whether or not they are treated in compliance to the guidelines resulting in better outcomes than the actual clinical decision. We include a detailed discussion about the assumptions made in constructing the BN and we highlight why such a BN can serve as an AI-based clinical decision support system particularly when there is need for further patient stratification.

## 1 Introduction

Hepatocellular carcinoma (HCC) is the third leading cause of cancer death worldwide (Huang et al., 2023) with a mortality rate of 96%. Thankfully there are an increasing number of treatment options available, while techniques such as trans-arterial chemoembolization (TACE) and selective internal radiation therapy (SIRT) are increasingly deployed as curative treatment options (Horvat et al., 2022). Patient management guidelines are widely used: in Europe these are predominantly the Barcelona Clinic Liver Cancer(BCLC) guideline (Reig et al., 2022); and the European Association for the Study of the Liver (EASL) guideline (European Association for the Study of the Liver, 2015). Recommendations for treatment modalities are usually based on BCLC cancer staging (Reig et al., 2022). Treatment options are based on assigning a patient to a broadly defined population subgroup. A clinician faces at least two challenges when applying such a guideline, especially as treatment decisions often need to be made quickly. First, the clinician needs to combine such a broad population subgroup guideline with specific information about the individual patient. Second, access to treatment modalities, medical compensation mechanisms, and local expertise/resource allocation are important factors that vary from site to site, complicating compliance with guidelines. In practice, treatments are personalised and indeed, there are situations where the recommended option does not align with jurisdictional treatment guidelines.

Bayesian networks (BNs) offer a way to combine guideline-specified standardized care with precision medicine. They do this by enabling answers to detailed “what-if” questions (technically: counterfactual reasoning) from the clinician. This study illustrates the utility of Bayesian network counterfactual reasoning by showing how a clinician may estimate potential survival time post treatment on a case-by-case basis. Causal inference with Bayesian networks is an AI method in which observational data is used to predict the likely outcome of an intervention. To be specific, we discuss the benefits and limitations of the approach in the context of implementation with the BCLC guideline. Technically, counterfactual reasoning is a form of probabilistic inference that is based on joint probability distributions and Bayesian statistics. We show how it is possible to (a) explicitly denote the influences between variables in the form of a Bayesian network (BN), (b) incorporate prior knowledge about unobserved variables to preserve individual uncertainties in the form of a probabilistic distribution, (c) perform interventions on specific variables to effect the specific counterfactual scenario, and (d) estimate potential treatment effects through the computation of posterior probabilities (Molak, 2023). Taken together, this enables assessment of individualised treatment effects and potentially serves as a virtual control or digital twin (Strayhorn, 2021).

## 2 Material and methods

### 2.1 Data preparation

A total of 190 patients’ data were curated from the open-source dataset Newcastle PLC (primary liver cancer) (Geh et al., 2022). We included only those cases that received liver cancer care before the possibly confounding effects of COVID-19 (patients were treated between March 2019 and February 2020). Descriptive statistics for patient demographics are summarized in Table 1. Metrics selected for further analysis include: tumour size (*Size*), HCC BCLC Stage (*BCLC_stage*), performance status (*PS*), treatment groups (*Treatment_grps*), elapsed time between the multidisciplinary meeting and time of first treatment (*T_MDM_first_treatment*) — i.e., patient’s waiting time, survival time following the multidisciplinary meeting (*Survival_fromMDM*), whether the patient is alive or dead (*Alive_Dead*). Some values were missing from the medical records, in which case they were filled according to the following strategy: there were 8 (4%) missing values for *Size* which were filled with the population mean; there were 3 (1.6%) missing values for *PS*, which were left “unknown”; there were 116 (62%) missing values for the metric *T_MDM_first_treatment*, which were imputed using Bayesian exact inference (Scutari, 2024). Descriptive statistics and data cleaning were performed using R (R Foundation, https://www.R-project.org/).

**TABLE 1:** Patient demographics.

Characteristic	N = 190
Age	71 (10)
Sex	
Female	44 (23%)
Male	146 (77%)
Cirrhosis_Yes	134 (71%)
Surveillance_Yes	75 (39%)
Aetiology	
Alcoholic Liver Didease	62 (33%)
Autoimmune	8 (4.2%)
Chronic hepatitis B	3 (1.6%)
Chronic hepatitis C	20 (11%)
Heamochromatosis	6 (3.2%)
Non-Alcoholic Fatty Liver Disease	69 (36%)
Unknown	22 (12%)
Present mode	
Incidental	80 (42%)
Surveillance	64 (34%)
Symptomatic	46 (24%)

### 2.2 Pathway analysis

The BCLC HCC management flowchart (Braunwarth et al., 2018) was adopted as the guiding principle to support judgement about whether each case in the dataset was treated in compliance with the guidelines. For the dataset, we considered the PS, the BCLC staging, solidary tumour size, and actual received treatment options. Our analysis investigates: (a) the proportional difference between the BCLC compliance versus non-compliance; and (b) which specific decision point tends to exclude a larger number of patients. Treatment compliance is created as a binary metric and combined into the larger dataset for the construction of Bayesian network and counterfactual inferences.

### 2.3 Construction of Bayesian networks

Bayesian networks and the corresponding probabilistic inferences were performed using the python library PyAgrum (Ducamp and Wuillemin, 2020). The metrics chosen were treatment *Compliance*, *Treatment_grps*, *T_MDM_first_treatment*, and *Survival_fromMDM*. The directed acyclic graph required for a BN was generated based on the assumption that the treatment a patient receives depends on whether or not the decision is to treat in compliance with the BCLC guideline. Both the type of treatment (*Treatment_grps*) and the waiting time (*T_MDM_first_treatment*) affect survival time (*Survival_fromMDM*). Parameter learning used the complete dataset derived from the data preparation phase. To avoid the “zero-probability” problem, we applied a smoothing prior with 0.001 weighting (Needham et al., 2007). We applied a smoothing prior with weighting 0.001 because, on the one hand it allows us to mitigate the so-called “zero probability” problem which inevitably derives from the limited size of the training data but on the other hand give highest weighting to real-world data, preserving the beneficial effect across different treatments. More discussion of the specific weighting factor 0.001 is given in the Discussion section. To allow individual variations and the explicit specification of personalization, two unobserved variables (Us, Ut) were generated. Us is an unobserved variable that affects *Survival_fromMDM* while Ut is the unobserved variable that affects *T_MDM_first_treatment*.

Note that both waiting time and survival length are discretised as integer values. This is a consequence of the design of the pyAgrum software package. The maximum survival time is capped at 40 months since this aligns with the original censoring of the Newcastle PLC (primary liver cancer) dataset. We chose to impute missing values instead of discarding samples because such cases tend to be patients who received supportive care. For cases received supportive care, in practice, clinical sites often do not record the waiting time because there is no active treatment to wait for. In fact, we previously trained the Bayesian network just with cases who have observed waiting time (*T_MDM_first_treatment*) and that lead to under-representation of the supportive care cases. We wanted to include this set of patients because we consider that they are a group who can benefit from counterfactual reasoning.

### 2.4 Counterfactual inferences

Counterfactual (“What if”) inferences follow three steps, which are known formally as Abduction, Action, and Prediction. **Abduction** refers to the construction of a hypothetical (“counterfactual”) situation that describes a specific scenario for that particular patient. This allows us to preserve an individual case’s bespoke profile. Technically, it is where the unobserved variables (Us, Ut) come into play to “absorb” a state of virtual reality that will represent a given patient. **Action** means to carry out (“do”) an action such as imposing a specific value for one or more variables. For example, we can change the waiting time for a treatment, or we can change the treatment from *non-specified* to *resection*. Technically, Action is based on the ***do***-operator introduced by Pearl and Mackenzie (2018). Finally, **Prediction** assumes that the Action step has been effected and then calculates the posterior probabilities for the target variable (e.g., survival time). This enables estimation of the most likely outcome using the hypothetical situation created in the Abduction step (Pearl and Mackenzie, 2018; Saha and Garain, 2022).

**FIGURE 1:**
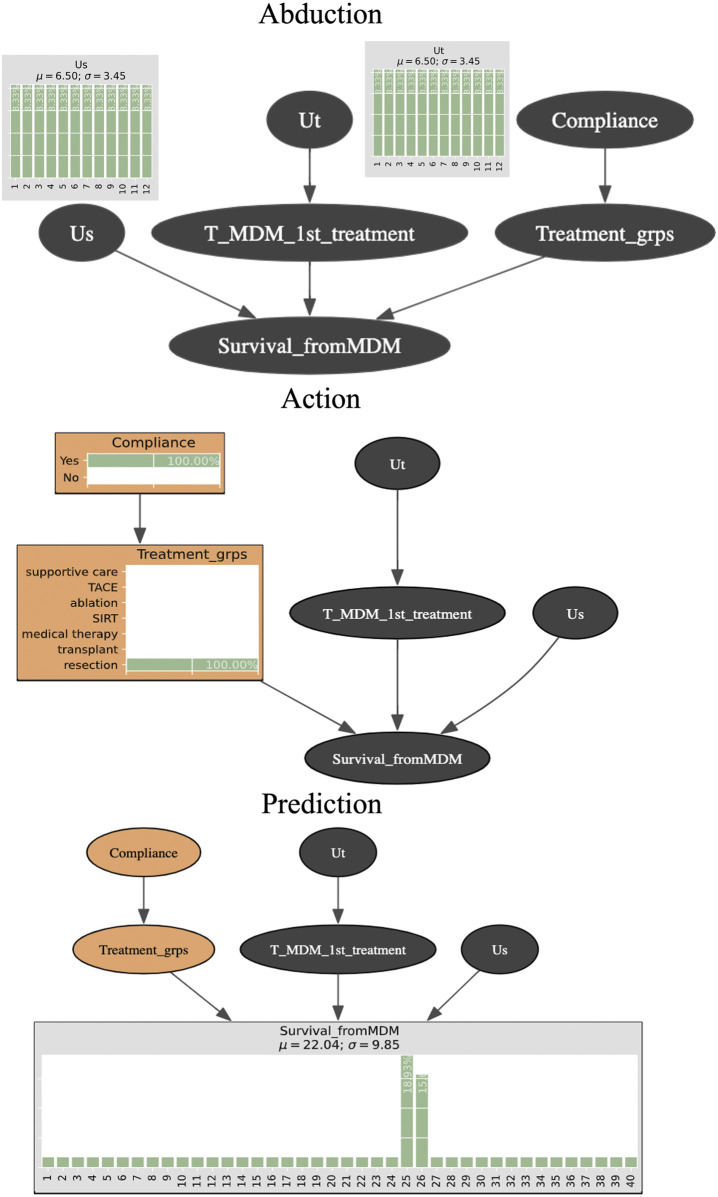
Schematic representation of performing counterfactual reasoning with Bayesian networks.

Figure 1 is a schematic of the three-step process for counterfactual inference and Table 2 shows the algorithm. We assume that the unobserved variables Us and Ut in **Abduction** range from 1 to 12 months since both are the parent nodes for time-related variables. Since they are “unobserved”, there is no prior knowledge about Us and Ut and so they are specified by uniform distributions. We intervene with a counterfactual (“what if” question) (*Compliance* = Yes, *Treatment_grps* = resection) during the **Action** step. The quantity of interest we seek to compute is the posterior probability for each level in the node *Survival_fromMDM*. **Prediction** is made by obtaining the survival time that is the maximum likelihood (for example, 25 months of 18.93% posterior probability).

**TABLE 2:** Algorithm for counterfactual inference.

Algorithm counterfactual inference
**Abduction** for i = 1, 2, 3, …,N **infer** $P\left(\left.Us,Ut\;\right|X_{i}=X_{i}^{obs}\right)$ ${Us}^{\prime },{Ut}^{\prime }⟵P\left(Us,\left.Ut\right|X_{i}=X_{i}^{obs}\right)$ **return** $Us^{\prime },Ut^{\prime }$ **modify** BN with $Us^{\prime },Ut^{\prime }$ ${BN}^{\prime }⟵P\left(X_{i}^{obs}\left({Us}^{\prime },{Ut}^{\prime }\right)\right)$ **return** ${BN}^{\prime }$
**Action** **intervene** on ${BN}^{\prime }$ ${BN}^{counter}⟵\;{BN}^{\prime }\left(do\left(X_{j}=X_{j}^{unob}\right)\right)$ **return** ${BN}^{counter}$
**Predict** **quantity** ${S\left(X_{j}^{unob}\right)\;from\;BN}^{counter}$ $S\left(X_{j}^{unob}\right)\;⟵\;P\left({Xi}_{X_{j}=X_{j}^{unob}}\left({Us}^{\prime },{Ut}^{\prime }\right)\right)$ **return** $S\left(X_{j}^{unob}\right)$

The following three questions were formulated to illustrate these applications of counterfactual reasoning:

1. What would the survival time be if the patient had been treated in compliance with the guideline? $S_{C\;}\left(x\right)$

2. What would the survival time be if the patient had to wait longer to receive treatment? $S_{T\;}\left(x\right)$

3. What would the survival time be had the patient been treated with other treatment options? $S_{Y\;}\left(x\right)$

In these three questions S(x) stands for months of survival after the multidisciplinary meeting assessed the case of patient x. C stands for the binary: whether the HCC patient x was or was not treated in compliance with the BCLC guideline. T stands for the time between the multidisciplinary meeting and the patient receiving their first treatment (i.e., the patient’s waiting time). Y stands for the available treatment options. In this particular dataset, Y includes liver resection, liver transplant, transcatheter arterial chemoembolisation (TACE), selective internal radiation therapy (SIRT), ablation, medical therapy, supportive care.

## 3 Results

### 3.1 Pathway analysis

**FIGURE 2:**
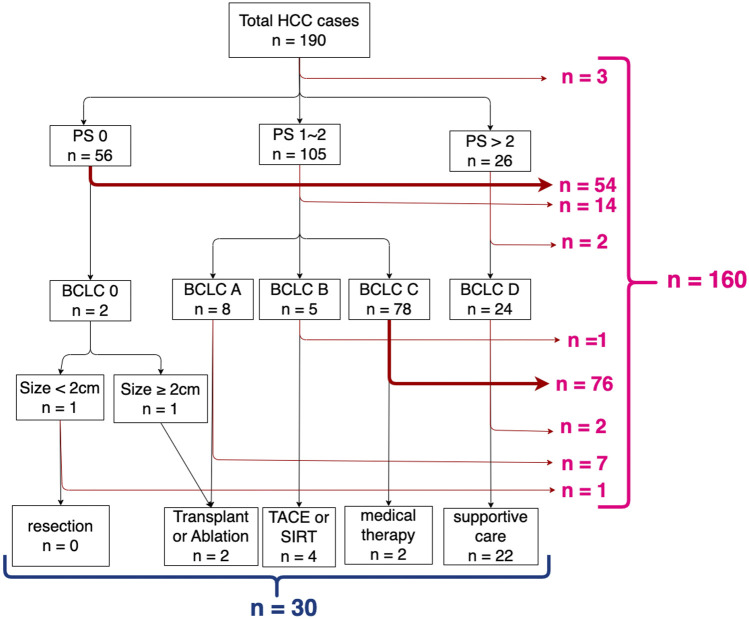
Results of pathway analysis mapping decision points (performance status, BCLC stage, and tumour size) according to the BCLC HCC management guideline.

Figure 2 shows the results of pathway analysis. Overall, 160 (84%) of the 190 patients were ***not*** treated in strict compliance with the BCLC HCC management guideline, leaving just 30 (16%) who were. Of the 160 “non-compliant” cases, the decision point at which there was greatest departure from compliance was at the second level decision point, where 76 (of 78 cases) with *BCLC = C* did not proceed to *medical therapy*. The second largest number (n = 54) occur at the first level decision point, *PS = 0 to BCLC = 0*. The remaining non-compliant cases are not discussed further because of the small numbers of patients in each case.

### 3.2 Counterfactual inference for 10 selected cases

We selected 10 cases to illustrate counterfactual “what if” reasoning. The cases highlight where Bayesian network counterfactual inference could add most to conventional use of the BCLC treatment pathway. We identified 2 decision points that corresponded to the greatest number of dis-concordance to the BCLC recommended pathway. The first was from BCLC = C to medical therapy, the other is from PS = 0 to BCLC = 0. Five cases were randomly drawn from each of these two decision points.

In more detail, we chose 5 cases (Cases1 to 5) that were excluded at the first decision point (*PS = 0 to BCLC = 0*). The remaining 5 cases (Cases 6 to 10) were non-compliant at the second decision point (*BCLC = C to medical therapy*). We performed BCLC-compliant counterfactual treatment (as summarized in Table 3), which were resection for Cases 1 to 5 and medical therapy for Cases 6 to 10 respectively. We find that in each of the ten cases, had the patient been treated in strict compliance to the BCLC guideline, they would have had a shorter survival time.

**TABLE 3:** Survival time comparison between actual treatment and BCLC-compliant treatment.

Case	Actual treatment	BCLC-compliant treatment
Case1	TACE (35 months)	resection (25 months)
Case2	transplant (32 months)	resection (25 months)
Case3	ablation (30 months)	resection (25 months)
Case4	TACE (28 months)	resection (25 months)
Case5	medical therapy (27 months)	resection (25 months)
Case6	SIRT (29 months)	medical therapy (17 months)
Case7	TACE (28 months)	medical therapy (17 months)
Case8	resection (25 months)	medical therapy (17 months)
Case9	supportive care (25 months)	medical therapy (17 months)
Case10	ablation (23 months)	medical therapy (17 months)

•Note that the waiting time are different between each patient but are kept the same within the same patient’s actual and counterfactual queries.

Additional counterfactual queries were developed for these 10 cases to take into consideration their demographic information and waiting time. Table 4 summarizes the results. The rows that are indicated with prime notation are the counterfactuals. In the right-most column, cases 1, 2, 4, 6, 7, 9, 10 had better outcomes whereas 3, 5, and 8 did not.

**TABLE 4:** Further counterfactual inferences for 10 selected cases.

Case	Age	Sex	Aetiology	Cirrhosis	Tumour present mode	Size	PS	BCLC stage	Waiting time	Treatment	Survival time
Case1	73	M	NAFLD	Yes	incidental	21	0	A	3	TACE	35
Case1′									4	resection	25
Case2	43	M	ALD	Yes	surveillance	7	0	A	20	transplant	32
Case2′									4	ablation	26
Case3	61	F	HCV	Yes	surveillance	13	0	A	4	ablation	30
Case3′									20	transplant	32
Case4	82	M	NAFLD	No	symptomatic	18	0	A	11	TACE	28
Case4′									7	SIRT	22
Case5	48	M	HCV	No	surveillance	10	0	C	3	medical therapy	27
Case5′									6	TACE	31
Case6	72	M	heamochro-	No	symptomatic	47	1	C	7	SIRT	29
Case6′			matosis						1	medical therapy	17
Case7	78	F	NAFLD	Yes	surveillance	17	2	C	7	TACE	28
Case7′									4	ablation	26
Case8	77	M	NAFLD	No	incidental	53	1	C	4	resection	25
Case8′									3	TACE	31
Case9	73	M	NAFLD	Yes	surveillance	6	2	C	2	supportive care	25
Case9′									3	SIRT	23
Case10	61	M	ALD	Yes	surveillance	12	1	C	8	ablation	23
Case10′									6	medical therapy	17

1: Note that the counterfactual inference result for each case is labelled with a prime notation (′). Therefore, the variation of Case1 is labelled as Case1′.

2: Waiting time and Survival time are measured in month(s).

3: NAFLD: non-alcoholic fatty liver disease; ALD: alcoholic liver disease; HCV: hepatitis C virus; TACE: transcatheter arterial chemoembolisation; SIRT: selective internal radiation therapy.

### 3.3 Counterfactual inference for Case1 and Case8

To illustrate how Bayesian network enable personalised assessment we now examine counterfactual inference in Case 1 that became non-compliant at the first decision point. The original profile of Case 1 was:

• $C_{factual}\left(Case1\right)=No$ – that is, Case1 was not treated in compliance with the BCLC guideline.
• $T_{factual}\left(Case1\right)=3\;months$ – that is, the waiting time for Case1 to receive treatment was 3 months.
• $Y_{factual}\left(Case1\right)=TACE$ – that is, the treatment that Case1 received was TACE.
• $S_{factual}\left(Case1\right)=35\;months$ – that is, the survival time for Case1 was 35 months.

The three counterfactual queries we made for Case1 were as follows:

1. *What would Case 1’s survival time have been be if he had been treated in compliance with the guideline?* We find that $S_{C=Yes\;}\left(Case1\right)\approx 25\;months$ (that is, 10 months less than the actual survival time).
2. *What would Case 1’s survival time have been if he had been required to wait longer to receive treatment?* We find that $S_{T=4\;}\left(Case1\right)\approx 8\;months$

3. *What would Case1’s survival time have been had he received other treatment options?* We find:

$$S_{Y=resection\;}\left(Case1\right)\approx 25\;months$$

$$S_{Y=transplant}\left(Case1\right)\approx 11\;months$$

$$S_{Y=ablation}\left(Case1\right)\approx 32\;months$$

$$S_{Y=SIRT}\left(Case1\right)\approx 12\;month$$

$$S_{Y=medical\;therapy}\left(Case1\right)\approx 17\;months$$

$$S_{Y=supportive\;care}\left(Case1\right)\approx 1\;month$$

Similarly, we examine Case 8 who was excluded during the second decision point. The profile of Case 8 is as follows:

• $C_{factual}\left(Case8\right)=No$ – that is, Case 8 was not treated in compliance to the BCLC guideline.
• $T_{factual}\left(Case8\right)=4\;months$ – that is, the waiting time for Case 8 to receive treatment was 4 months.
• $Y_{factual}\left(Case8\right)=resection$ - that is, the treatment that Case 8 received was resection.
• $S_{factual}\left(Case8\right)=25\;months$ - that is, the survival time for Case 8 was 25 months.

The counterfactual queries for Case 8 were as follows:

4. *What would Case 8’s survival time have been be if he had been treated in compliance with the guideline?* We find that $S_{C=Yes\;}\left(Case1\right)\approx 17\;months$ (that is, 8 months less than the actual survival time).
5. *What would Case 8’s survival time have been if he had not needed to wait as long to receive treatment?* We find that $S_{T=3\;}\left(Case8\right)\approx 31\;months$

6. *What would Case 8’s survival time have been had he received other treatment options?* We find:

$$S_{Y=transplant}\left(Case8\right)\approx 11\;months$$

$$S_{Y=ablation}\left(Case8\right)\approx 32\;months$$

$$S_{Y=TACE\;}\left(Case8\right)\approx 31\;months$$

$$S_{Y=SIRT}\left(Case8\right)\approx 12\;month$$

$$S_{Y=medical\;therapy}\left(Case8\right)\approx 17\;months$$

$$S_{Y=supportive\;care}\left(Case8\right)\approx 1\;month$$

### 3.4 Validation

Bayesian networks comprise two parts: a graphical structure that expresses the relationship between the relevant variables, and the quantitative aspect, which includes the joint probability distributions. In this case, the graphical structure was developed by way of consultations with the clinician (KH) and three senior scientists at our institution. There was total agreement on the structure of the network. As regards the quantitative aspect of the BN, we transformed the trained network into a classifier and used receiver operator curve analysis with a 70-30 train test split to assess accuracy. This yielded an AUC = 0.64 for both the training and testing data.

## 4 Discussion

### 4.1 Pathway analysis

A remarkably large percentage (84%) of the 190 cases were ***not*** treated strictly in concordance with the BCLC 2022 guidelines. One may object that our analysis should have been based on BCLC 2018 guideline (Forner et al., 2018) since that is the version from which the retrospective data were collected. In that case, metrics such as solitary tumour size, PS, and BCLC staging have the same thresholds as for the 2022 guideline. The key difference is that all these metrics would be considered at the same decision point. Therefore, although it would not affect the overall proportion of compliance and non-compliance, it would reduce our ability to expand the decision points into three levels and so would not enable us to understand which decision point is ruling people out for eligible treatments.

### 4.2 Counterfactual inference for Case 1 and Case 8

At the time it was discovered, the solitary tumour size for Case 1 was 2.8 cm and the diagnosis confirmed as BCLC stage A and performance status 0. This patient could be treated with resection according to the BCLC guideline. However, Case 1 is a 73-year-old male with non-alcoholic fatty liver disease (NAFLD) and liver cirrhosis. An understandable and reasonable decision by the clinician was to prescribe TACE. Yet, the clinician would (and, to be in compliance with the Guideline should) reasonably ask “If I were to treat this patient according to the guideline, would the outcome be better?”

We observed that for Case 1, each of the three counterfactual queries (except for being treated with supportive care) would have resulted in a shorter survival time than that actually achieved (35 months). This illustrates that despite not following the management guideline, it is likely that the best decision was made for Case1.

Case 8 is a male NAFLD patient aged 77. Demographically, Case 8 is similar to Case 1 though at the time the tumour was discovered, its size was already 5.3 cm and his diagnosis confirmed as BCLC stage C and performance status 1. According to the BCLC guideline, this patient should have been treated with medical therapy. However, Case 8 did not present signs of liver cirrhosis. The clinician possibly considered this fact and prescribed resection instead.

Counterfactual reasoning showed that had Case 8 been treated with medical therapy, he would have had an 8-month shorter survival time. Once again, despite not following the management guideline, it is likely that a better decision was made for Case 8.

Counterfactual queries such as these are of particular value during the multidisciplinary (tumour board) meetings because they inform “what-if” scenarios. Moreover, one can compute the joint posterior probability of all these combined counterfactual situations with the same Bayesian network model, for example:

$$S_{C=Yes,T=4,Y=resection}\left(Case1\right)\approx 25\;months$$

$$S_{C=Yes,T=3,Y=medical\;therapy}\left(Case8\right)\approx 27\;months$$

This machinery of counterfactual inference provides a scheme for controlling known confounders and can be applied to enhance trust and the performance of AI-supported decision systems (Saha and Garain, 2022)

Interestingly, we note that had Case 8 been treated with TACE (while keeping all other variables unchanged), he would likely have had a longer survival time (31 months). As noted in the introduction, increasing evidence has shown local regional therapy such as TACE can be used as a curative treatment or down-staging strategy (Horvat et al., 2022). Bayesian network based counterfactual inference as developed in this work suggests that it could, at the very least, flag an opportunity during the multidisciplinary meeting which may otherwise be missed.

### 4.3 Counterfactual inference for 10 selected cases

Counterfactual inferences should be compared against the actual case for an individual instead of aggregated summary because each case would potentially have an alternative treatment that is specific to their particular circumstances. We found that despite not following the BCLC guideline, better decisions were made for all cases. With further counterfactual inferences where we intervened on both the waiting time (*T_MDM_first_treatment*) and treatment option (*Treatment_grps*), we found that in 3 of the 10 cases there could have been increased survival time, while 7 cases would have shortened survival time had they been treated with these respective counterfactual scenarios. The Bayesian network that we built is able to infer both positive and negative outcomes.

One may also note that there will be situations where counterfactual inference is not obtainable. In our example of HCC, when a 40-year-old patient’s tumour is diagnosed at an early stage (*Size < 2 cm, BCLC = 0, PS = 0*) and without signs of liver cirrhosis, a clinician would likely not prescribe supportive care, rather opt for resection. Therefore, the counterfactual inference with the configuration (*Size < 2 cm, BCLC = 0, PS = 0, Treatment_grps = supportive care*) will likely not be obtainable because it is rarely presented in the parameter learning phase.

### 4.4 Discussion of assumptions

Due to the counterfactual nature where a potential outcome is unobserved, it is challenging to verify our estimations of counterfactual queries. In such situations, one would aim to reduce, as much as possible, bias introduced into the system by assessing the plausibility of assumptions made in the process of constructing Bayesian networks for causal inference. Below we detail the assumptions made and why in some cases those assumptions would not be justified.

In this study we assumed that the waiting time (*T_MDM_first_treatment*) is independent of whether or not the patient will be treated in compliance with the BCLC guideline *(Compliance*). In other words, we assumed that there are no unobserved confounding variables between these two metrics. In causal estimation, this is often called the “*ignorability assumption*” (Feuerriegel et al., 2024). More research on this point is needed because it is possible that the decision to **not** treat in compliance with the guideline is because local expertise in alternative options is more readily available for the specific clinical site. Consequently, the patient does not need to wait as long to receive treatment.

Note also that in this study we did not specify unobserved variables that influence the node *Compliance* and subsequently *Treatment_grps*. This is because, as stated in (Saha and Garain, 2022), it is not required to know an intervened variable’s parent nodes and corresponding noise variable (i.e., unobserved variable) during abduction if we are given the observed value for the variable to be intervened. Note that intervention here means the **Action** step of counterfactual inference with ***do***-operator. There could be situations where obtaining the unobserved variable for *Compliance* node is necessary. For example, if the waiting time (*T_MDM_first_treatment*) and *Compliance* node are structurally connected and we intervene solely on the *T_MDM_first_treatment* node, the state of unobserved variable for *Compliance* node is necessary for counterfactual inference.

We also assume that the counterfactual outcome for the patient to receive different treatment options is identifiable from the population observational data. In causal estimation, this is called the “*identifiability assumption*” (Feuerriegel et al., 2024). In a real clinical setting, even though a clinician, considering a patient’s specific circumstances, would not consider all seven possible treatment options (in reality, two or three options is more likely), the ability to estimate ***all*** potential treatment outcomes supports the choice of treatment, including combination treatments. In the case of HCC, tumour down-staging with TACE/SIRT following curative resection is becoming more commonly used (Sangro and Salem, 2014). In our experience with this study, potential outcomes from all treatment options can be identified from the Bayesian networks with counterfactual reasoning. However, estimating the potential outcome for SIRT was particularly challenging. This is primarily because the SIRT option is under-represented in the training dataset (collected in 2019), reflecting the fact that SIRT is a relatively recent therapy option. As of the latest BCLC 2022 guideline, no-evidence based recommendation is made for using SIRT for BCLC-B and C category; as for BCLC-0 and BCLC-A category, SIRT can be used when TACE is not available.

We also assume that the outcome (in this case survival time) does not depend on the treatment assignment of other patients, and there is no hidden variation in the effect of the treatment across different settings or populations. This is called the “*stable unit treatment value assumption*” (SUTVA) (Feuerriegel et al., 2024). The former clause holds true because each patient is treated on a case-by-case basis. As for the latter clause, we can only generally accept that this assumption will hold because our study population were all referred to the same centre, namely the Newcastle upon Tyne NHS Trust and so the available expertise was likely mostly unchanged. For most HCC therapies, the intent is to remove the tumour in a single session. One can therefore consider 100% removal as a “stable unit”. Indeed, it could be possible that the same procedure (e.g., resection) performed by different surgeons on different patients could lead to variation of treatment effects. In the case of medical therapy or chemotherapy, where the anti-cancer effect is applied over time, the unit of treatment value might not be considered stable. More research is needed to consider this assumption.

Guidelines develop, often substantially, over time. Specifically, there were substantial changes between BCLC 2022 and BCLC 2018. The major difference between them is that cancer staging, performance status, and tumour size are considered at a single timepoint in BCLC 2018; but in separate steps in BCLC 2022. Applying our method to BCLC 2018 would impact the identification of the point at which BCLC recommendations may need further stratification. This would have impacted the choice of cases selected for analysis, but not the overall thrust of the paper.

Finally, and technically, recall from Section 2.3 that we imposed a smoothing prior weighting of 0.001. It is generally accepted that a smoothing prior helps mitigate the “zero probability” issue that derives from relatively small training sets. Nevertheless, such smoothing priors intrinsically bias the joint probability distributions. To assess the impact this may have in the current application, we systematically varied the smoothing prior from 0.001 to 1.0 while retaining the same training data. We found that the main change was to the node *Survival_from_MDM* (i.e., survival time) where the predicted survival times (months) became more equal to each other. Conversely, when we reduced the smoothing prior from 0.001 by factors of 10 (0.0001, 0.00001, …) this made no change to the associated distributions. For this reason, we chose 0.001, which means that the training data (real-world data) is weighted as 99.9%.

### 4.5 Limitations

There is inevitably selection bias particularly due to the fact that patients who are diagnosed with a liver tumour will almost always receive treatment. As a result, we could not assume that for each possible combination of patient characteristics, we can observe both treated and untreated patients [this is called the “*positivity assumption*” (Feuerriegel et al., 2024)]. On the other hand, our training data could exhibit zero conditional probabilities for certain combination of patient characteristics, treatments, and survivals. This actually reflects the difficulties of clinical data gathering rather than there being no chance of a certain situation happening. To mitigate such scenario of “zero-probability” (Needham et al., 2007) and enable counterfactual inference, we applied a smoothing prior. In particular, we chose a low weighting (0.001) to reduce the bias by giving the actual training dataset a relatively 1000 times more importance than the artificial prior. This choice is arbitrary, and we appreciate the fact that this can still introduce bias. Therefore, for each counterfactual query, we explicitly specify the most appropriate alternative treatment option that is largely agreed clinically. Further research can adopted propensity score matching to tackle the positivity assumption and mitigate for the necessity of priors.

This study is based on a sample size (n = 190) and 62% of the missing values for *T_MDM_first_treatment* (i.e., patient’s waiting time) were imputed. From a machine learning point of view, 190 would be considered tiny; but from the standpoint of clinical practice in primary liver cancer it is substantial. When imputing the missing values for patient’s waiting time, we assumed that the missing values can be estimated from observational data (this is called the missing-at-random assumption) (Daly et al., 2011). We use Bayesian exact inference to account for the influence from both the nodes *Treatment_grps* and *Survival_fromMDM.* This way the imputed values likely sit between the possible range in our training data, more closely reflecting reality. Even at first-rate clinics, it is inevitable that there are missing values and there could be deeper reasons as to why patient’s waiting time tend to be omitted for recording, which is an issue that is not likely to be improved until systematic change at the institutions. In this study, we had to work with what was available. We are currently developing a project that has almost 5 times as many patients, and more outcome values (e.g., progression free survival, time to recurrence). This will both enable a comparison against the current pilot study and improve confidence in such a methodology.

Abduction is a process to allow personalized scenarios to be preserved. Mathematically, this is done by introducing unobserved variables to temporarily “absorb” a certain joint probability distribution for an individual case. When proceeding to the action step with counterfactual evidence, we sample on this “newly absorbed distribution” and calculate posterior probabilities for the outcome node. In this study, we introduced two unobserved variables (Us, Ut). Note that Us and Ut are “unobserved” but “not latent”, meaning that they are not just random variables, but we have a certain expectation on their effects of *Survival_fromMDM* and *T_MDM_first_treatment*. It is perhaps more suitable to describe Us and Ut as “class of unobserved variables” so the probability they represent is joint probability distributions of the class of unobserved variables. Because there could likely be many factors influencing the survival and waiting time, inference can quickly become complicated, which is why currently we assume uniform distributions for both Us and Ut. Our future studies will aim to expand the Bayesian network by collecting more influencing and outcome metrics and explicitly specify values during the abduction phase.

Finally, the data used in this study comes from a single clinical site. Nevertheless, the methodology outlined in this paper is generally applicable both to other HCC clinical sites and to other pathologies. As well, combining data from a plurality of sites would further reduce the impacts of other assumptions, for example, the specific value of the smoothing prior. We would always expect centre-specific practices to influence the results of counterfactual reasoning. This is also why we explain where the stable unit treatment value assumption (SUTVA) could be implausible. An example could be where one centre can access the latest development of selected internal radiation therapy (SIRT), the treatment effect will be different from the centre where SIRT is less accessible, which is currently what we observed from the Newcastle dataset.

### 4.6 Clinical Significance

We stress that this study is **not** intended as a criticism of clinicians! Quite the contrary, the study further emphasises the complexity of real-world clinical decision making and reflects the nuances that clinicians face daily regarding treatment decisions. As seen from the pathway analysis, a larger proportion of patients did not fall into the treatment trajectory of the BCLC guideline. This is usually not a problem unless allegations of clinical negligence occur. Instead of passively hoping such events do not happen, this study attempts to actively demonstrate the possibility of supporting a clinician’s judgement on potential outcomes by using counterfactual analysis. Additionally, we identified two decision points that exclude a large number of patients (*PS = 0 to BCLC = 0*) and (*BCLC = C to medical therapy*). This highlights the need for further or more flexible stratification strategies. To our knowledge, this study is the first that demonstrates specific counterfactual reasonings for HCC population in those cases.

## 5 Conclusion

This study illustrates counterfactual inference of individual treatment effects for a group of HCC patients based on real-world data and the current BCLC guideline. Tools such as Bayesian networks can be used to support treatment decisions and potentially serve as an intermediate judgment device when there is need for more flexible stratification of patient populations. Our methodology is broadly generalizable, in particular to areas of clinical medicine for which there are multiple treatment options, and which are subject to internationally agreed treatment guidelines.

## Data Availability

Publicly available datasets were analyzed in this study. This data can be found here: All data created during this research are openly available at https://doi.org/10.25405/data.ncl.19590727.v1.
